# Allergy and Sensitization during Childhood Associated with Prenatal and Lactational Exposure to Marine Pollutants

**DOI:** 10.1289/ehp.1002289

**Published:** 2010-06-20

**Authors:** Philippe Grandjean, Lars K. Poulsen, Carsten Heilmann, Ulrike Steuerwald, Pál Weihe

**Affiliations:** 1 Institute of Public Health, University of Southern Denmark, Odense, Denmark; 2 Department of Environmental Health, Harvard School of Public Health, Boston, Massachusetts, USA; 3 Laboratory for Medical Allergology, Allergy Clinic and; 4 Paediatric Clinic II, Copenhagen University Hospital, Rigshospitalet, Copenhagen, Denmark; 5 Department of Occupational Medicine and Public Health, Tórshavn, Faroe Islands

**Keywords:** allergy, breast-feeding, developmental toxicity, environmental exposure, immunotoxicity, methylmercury, polychlorinated biphenyls

## Abstract

**Background:**

Breast-feeding may affect the risk of developing allergy during childhood and may also cause exposure to immunotoxicants, such as polychlorinated biphenyls (PCBs), which are of concern as marine pollutants in the Faroe Islands and the Arctic region.

**Objectives:**

The objective was to assess whether sensitization and development of allergic disease is associated with duration of breast-feeding and prenatal or postnatal exposures to PCBs and methylmercury.

**Methods:**

A cohort of 656 singleton births was formed in the Faroe Islands during 1999–2001. Duration of breast-feeding and history of asthma and atopic dermatitis were recorded at clinical examinations at 5 and 7 years of age. PCB and mercury concentrations were determined in blood samples obtained at parturition and at follow-up. Serum from 464 children (71%) at 7 years of age was analyzed for total immunoglobulin E (IgE) and grass-specific IgE.

**Results:**

The total IgE concentration in serum at 7 years of age was positively associated both with the concomitant serum PCB concentration and with the duration of breast-feeding. However, the effect only of the latter was substantially attenuated in a multivariate analysis. A raised grass-specific IgE concentration compatible with sensitization was positively associated with the duration of breast-feeding and inversely associated with prenatal methylmercury exposure. However, a history of asthma or atopic dermatitis was not associated with the duration of breast-feeding, although children with atopic dermatitis had lower prenatal PCB exposures than did nonallergic children.

**Conclusions:**

These findings suggest that developmental exposure to immunotoxicants may both increase and decrease the risk of allergic disease and that associations between breast-feeding and subsequent allergic disease in children may, at least in part, reflect lactational exposure to immunotoxic food contaminants.

Exposures to marine contaminants are of much concern to populations that rely on seafood for their livelihood. Among main contaminants resulting in increased exposures, methylmercury and polychlorinated biphenyls (PCBs) share immunotoxic potentials (Belles-Isles et al. 2002; Bilrha et al. 2003; Heilmann et al. 2006). Immunotoxicity is of particular concern when the exposures happen during the development of the immune system. Important windows of vulnerability are the intrauterine and the early postnatal periods, when unique immune maturational events take place (Dietert 2008). Breast-feeding is thought to play an important role for the infant’s immune system development, but the evidence is equivocal in regard to the extent of possible protection against allergic disease (Bergmann et al. 2002; Kirsten 2009; Kramer et al. 2007; van Odijk et al. 2003).

As illustrated by studies on 2,3,7,8-tetrachlorodibenzo-*p*-dioxin (TCDD) (Kerkvliet 2009), an immunotoxicant may disrupt different immune maturational processes, depending upon the specific developmental timing of exposure (Dietert 2008). Because development of T-helper cell type 2 (Th2) functions is favored prenatally, whereas acquisition of Th1 functional capacities happens postnatally, the effects may depend on the age at peak exposure. For substances such as methylmercury, the peak exposure occurs during prenatal development, when the fetus shares the contaminant from the mother’s diet; human milk is not an important exposure pathway for this substance (Grandjean et al. 1995). However, lipophilic contaminants, such as PCBs, accumulate postnatally, so longer breast-feeding periods will result in higher body burdens in the child (Barr et al. 2006; Patandin et al. 1997).

Exposures to PCBs, methylmercury, and related substances are increased in the Arctic region (Belles-Isles et al. 2002; Bilrha et al. 2003; Heilmann et al. 2006), especially where marine mammals are part of the traditional diet. The prevalence of allergy in the Arctic has generally been assumed to be low (Krause et al. 2002b), although the degree of sensitization has been dramatically increasing toward the end of the last millennium (Krause et al. 2002a).

We carried out a prospective study of a birth cohort in the Faroe Islands, a North Atlantic fishing community with increased average dietary exposures to methylmercury and PCBs from pilot whale meat and blubber (Steuerwald et al. 2000). Extended breast-feeding is common in this community (Grandjean et al. 1994). Outcome parameters were total level of immunoglobulin E (IgE), grass-specific IgE, and occurrence of allergic disease.

## Methods

### Birth cohort and clinical examinations

In the Faroe Islands, a birth cohort was formed from consecutive spontaneous births during 1999–2001 (Heilmann et al. 2010). Informed consent and baseline data were obtained from a total of 656 mothers in connection with their singleton births. Obstetric variables, including birth date, birth weight, gestational age, parity, and maternal age, were obtained, as was information on maternal smoking and alcohol use during pregnancy; dietary history during pregnancy was obtained from approximately half of the mothers.

Detailed follow-up examinations were scheduled for the whole cohort at approximately 5 and 7 years of age. They included physical examination, blood sampling, and a maternal interview on the child’s current health and past medical history, including duration of breast-feeding (exclusive and total, in months). Occurrence of asthma and atopic dermatitis at the follow-up examinations was determined by a single pediatrician, who examined all the cohort children and interviewed the mother. Parental smoking at home and child care attendance were recorded, but family history was not explored because the focus of the study was on environmental chemical exposures.

The present report is based on the cohort members who were examined at 7 years of age and provided a blood sample sufficient for IgE and contaminant analyses. For the 76 cohort children who did not participate in the 7-year examination, the main reasons were decision to leave the follow-up study (*n* = 29), the child did not want to participate this time (*n* = 28), current residence abroad (*n* = 13), deceased child (*n* = 3), and miscellaneous (*n* = 3). For 67 of the children examined, a blood sample was not obtained, and in 49 cases insufficient serum was available. Overall, IgE results and clinical data were available for 464 cohort children (71% of original cohort).

The study protocol was approved by the ethical review committee serving the Faroe Islands and by the institutional review board at Harvard School of Public Health.

### Exposure assessment

Exposures to marine contaminants were assessed from analysis of biological samples obtained at the prospective clinical examinations. PCB exposure was determined from analyses of serum and milk, and methylmercury exposure from mercury analyses of whole blood and maternal hair (Heilmann et al. 2006). For PCB analysis, maternal serum was obtained at the last antenatal examination in the 34th week of pregnancy, and transition milk was sampled before the mother left the hospital (4–5 days after parturition). Cord blood and maternal hair for mercury analysis were obtained in connection with the parturition. Serum, whole blood, and hair were also obtained from the children at the time of the clinical examinations.

Serum analyses were conducted by gas chromatography with electron capture detection at the University of Southern Denmark (Heilmann et al. 2006). Milk analyses were performed by similar methodology at the Department of Environmental Health, State Agency for Health and Occupational Safety of Schleswig-Holstein, Germany (Schade and Heinzow 1998). To avoid problems with congeners not assessed and concentrations below the detection limit, a simplified concentration of the sum of PCBs (∑PCB) was calculated as the sum of congeners PCBs 138, 153, and 180 multiplied by 2 (Grandjean et al. 1995). Although the analysis included major PCB congeners and other persistent environmental chemicals, they were disregarded because of close correlations with ∑PCB. The ∑PCB concentrations were expressed in relation to the total lipid concentration. Because of the high correlation between ∑PCB concentrations in maternal serum and milk (*r* = 0.89), missing serum data (*n* = 152) were calculated from the milk result using the average ratio (1.13) between the two.

Mercury concentrations in whole blood and hair were measured by atomic absorption technique (Grandjean et al. 2003). Hair and blood concentrations correlated very well (*r* = 0.84 both at birth and 7 years of age). When blood results were missing (e.g., *n* = 30 for cord blood), the average ratio between the two (e.g., 4.25 to convert concentrations in maternal hair to cord blood) was used to estimate the blood concentration.

### Total IgE and anti-grass IgE assays

Because of the limited amount of serum available from the cohort children at 7 years of age, only total IgE and IgE specific to grass pollen (*Phleum pratense*, allergen code g6) were determined by the ImmunoCAP system (Phadia, Uppsala, Sweden) according to the manufacturer’s instructions. For the latter assay, allergen-specific units exceeding 0.35 kUA/L indicated sensitization. Grass pollen is ubiquitous and seems to be the most frequent sensitizing allergen in both Greenland and Iceland (Clausen et al. 2008; Krause et al. 2002b).

### Data analysis

Exposure parameters and total IgE concentrations were log-transformed to obtain normally distributed residuals with a homogeneous variance. Results were therefore expressed as geometric means and the interquartile range (25th to 75th percentiles). Comparisons were performed with independent-sample *t*-tests and correlation coefficients. Associations were further explored in linear regression models. Adjustment for covariates—i.e., sex, age, season of birth, preterm birth (34th through 36th week, *n* = 11), low birth weight (< 2,500 g, *n* = 4), maternal age, parity, maternal fish intake and smoking during pregnancy, parental smoking at home, daycare attendance, and the child’s body mass index—was then included in the model to ascertain whether they materially affected (> 10%) estimated effects of immunotoxicant exposure. For grass-specific IgE concentration and the duration of breast-feeding, which deviated from normal distribution also after transformations, Spearman’s nonparametric correlation coefficients and logistic regressions were applied. Statistical significance was assumed when *p* < 0.05 (two-sided). For all calculations, SPSS version 15 (SPSS Inc., Chicago, IL, USA) was applied.

## Results

Table 1 shows obstetric characteristics and overall exposure levels. The exposure biomarkers showed positive correlations, especially for PCB measured at different points in time (Table 2). The duration of breast-feeding showed a strong association with postnatal PCB exposure only.

**Figure 1:**
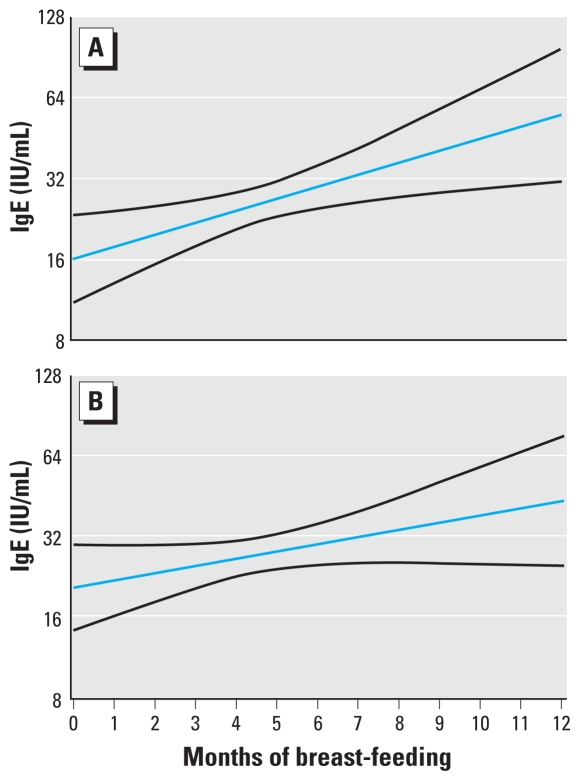
Total IgE concentration in serum (log scale) in 464 children 7 years of age from a Faroese birth cohort in relation to the duration of exclusive breast-feeding: unadjusted regression line with the 95% confidence interval (*A; p* = 0.003) and results after adjustment for the child’s concomitant serum PCB concentration (*B; p* = 0.11).

Table 3 shows the exposure data within tertile groups of the total IgE concentrations at 7 years of age. All correlation coefficients were rather small (*r* < 0.2), but the *p*-values suggest that some of the associations could not be ascribed to chance. The highly significant positive correlations with the serum PCB concentration at 5 and 7 years of age suggested that a doubling in PCB was associated with an increase in total IgE of about 18%. We found a weaker tendency in the same direction for methylmercury exposure, but in this case only for prenatal exposure. Duration of breast-feeding also showed a positive association with IgE: Each month of exclusive breast-feeding was associated with an average increase of total IgE of 12%. However, when adjusted for serum PCB at 7 years of age in a multiple regression analysis, the increase in IgE for each month of breast-feeding decreased to 6% (*p* = 0.13) (Figure 1); the regression coefficient for PCB decreased marginally to 17% in this analysis and remained statistically significant (*p* = 0.02). Maternal fish intake during pregnancy, as a measure of prenatal exposure to n-3 fatty acids, was not associated with the child’s total IgE concentration (*p* = 0.28). Other covariates did not materially affect these associations.

PCB exposure variables in cohort members with measurable grass-specific IgE > 0.35 kUA/L, consistent with allergic sensitization, did not differ from those observed in subjects with low or nondetectable concentrations (Table 4). However, the mean prenatal methylmercury concentration was lower in grass-sensitized children than in other children. Although exact IgE concentrations may be of limited clinical relevance, the Spearman correlation coefficient supported this negative association (*r*_s_ = −0.17; *p* < 0.001). We observed a tendency in the opposite direction regarding the total duration of breast-feeding (*r*_s_ = 0.11; *p* < 0.03). Mutual adjustment in a logistic regression analysis suggested that both factors were independently associated with grass-specific IgE.

Nine of the 464 children had a history of both asthma and atopic dermatitis; 26 had asthma only, and 51 had atopic dermatitis only. Total IgE concentrations and proportions of children with grass-specific IgE > 0.35 kUA/L at 7 years of age were increased in children with asthma or atopic eczema relative to nonallergic children (Table 5). Of the 39 children who showed increased grass-specific IgE, 8 had a history of asthma and 15 a history of atopic dermatitis; 3 had a history of both. Children with a history of atopic dermatitis had lower PCB exposures than did nonallergic children, most clearly reflected by the prenatal exposure levels. In contrast, children with a history of asthma had slightly higher PCB exposures than did nonallergic children, although the latter association could be due to chance. We observed no obvious associations with methylmercury exposures, although children with asthma had slightly higher mercury concentrations prenatally and at 5 years, compared with nonallergic children. We further explored these tendencies in logistic regression analyses with the diagnoses as the dependent variable. None of the covariates materially affected the unadjusted results shown in Table 5.

## Discussion

The strength of the present study is that a population-based birth cohort was followed prospectively with repeated assessment of exposures to marine contaminants for comparison with the allergy and sensitization status up to 7 years of age. Compared with other populations, average exposures to both PCBs and methylmercury were high and ranges of exposures were wide, thereby adding statistical power to the study. Almost 90% of the children participated in the examinations at 5 and/or 7 years of age, and we obtained IgE data at age 7 years from 71%.

As a main finding, serum PCB concentrations at 7 years of age were positively associated with total IgE concentrations. We observed a similar tendency for the prenatal methylmercury exposure, although this correlation could be due to chance. Longer duration of breast-feeding also appeared to predict a higher IgE concentration at 7 of age, but adjustment for the effect of PCB exposure reduced this association so that a chance finding could not be ruled out. For the grass-specific IgE concentration, the duration of breast-feeding again showed a positive correlation, now without a concomitant association with PCB. In addition, we observed an inverse association between grass-specific IgE levels and prenatal methylmercury exposure. Regarding clinical diagnoses, prenatal PCB exposures were inversely associated with a history of atopic dermatitis but showed a weak positive association with asthma. These diverse findings suggest that mechanisms for immunotoxicant effects for total and grass-specific IgE differ from those for asthma and atopic dermatitis.

Because we based the clinical assessment of atopic disease only on examinations at ages 5 and 7 years and on maternal interview, the present study cannot elucidate the possible role of immunotoxicants in the complex pathophysiological origins of these conditions. However, IgE concentrations have become routine clinical parameters in allergological diagnostics (Weinmayr et al. 2010). We chose total IgE as a marker of general IgE synthesis, and IgE specific to grass (*Phleum pretense*) as an important marker of sensitization, because grass pollen is ubiquitous and has previously been demonstrated to be the most common allergen in other North Atlantic environments (Clausen et al. 2008; Krause et al. 2002b). For grass pollen, we found 39 positives, corresponding to a sensitization rate of 8.3%, which is slightly lower than found in Western Greenland at 5–18 years of age; this difference is consistent with the lower age of our study population.

Markers of allergic reactions have previously been reported to be associated with a variety of environmental factors (Nagayama et al. 2007; Poulsen and Hummelshoj 2007; ten Tusscher et al. 2003). Sensitization reflected by a specific IgE is not necessarily governed by the same mechanisms as the ones determining total IgE level, as suggested by studies of parental smoking that showed associations in different directions for total IgE and skin test positivity (Strachan and Cook 1998). Among indications of immune dysfunction associated with increased exposures to PCBs and dioxins, mononuclear cells from cord blood showed decreased *in vitro* secretion of tumor necrosis factor-α after mitogenic stimulation; this cytokine is an important proinflammatory stimulant (Bilrha et al. 2003). Other exposure-related associations include differences in lymphocyte population ratios in peripheral blood from populations exposed to PCBs and related substances (Nagayama et al. 2007; ten Tusscher et al. 2003; Van Den Heuvel et al. 2002). Laboratory animal studies suggest that mercury compounds may induce autoimmune disease and increases in interleukin-4 (IL4) production and IgE levels in certain rodent strains (Nielsen and Hultman 2002). In human peripheral blood mononuclear cells *in vitro*, methylmercury concentrations of 100 μg/L were capable of inducing Th2 cytokine production, whereas γ-interferon production suppression occurred at 400 μg/L; in this model, mercury chloride stimulated increases in IL4 only at 1,000 μg/L (de Vos et al. 2007).

In the present study, mutual correlations between PCB and methylmercury concentrations were weak and did not prevent characterization of their differing associations with the immunology parameters. In contrast, individual PCB congeners and ∑PCB concentrations in serum correlated very closely with one another. Close correlation also occurs with other persistent organic pollutants, such as *p*,*p*′-dichlorodiphenyldichloroethene (*p*,*p*′-DDE) (Heilmann et al. 2006, 2010). Although we focused on the ∑PCB concentration as a reliable overall marker of lipophilic contaminant exposure, we were unable to assess possible immunotoxic effects of individual PCB congeners or associated pollutants, which commonly occur in seafood together with PCBs.

Regarding other seafood constituents, maternal n-3 fatty acid intake from fatty fish is thought to affect the development of her child’s immune system (Furuhjelm et al. 2009). In the Faroese fishing community, positive correlations occur between serum concentrations of n-3 fatty acids and PCBs, although the latter mainly originates from pilot whale blubber (Steuerwald et al. 2000). Although n-3 fatty acids were not measured in the present study, the absence of any association between maternal fish intake during pregnancy and the immune parameters examined would argue against any important confounding due to maternal n-3 fatty acid intake during pregnancy. In a wider sense, confounding from other risk factors would likely be limited in this Nordic population with relatively uniform living circumstances, also taking into account the increased average level and wide range of exposures to the seafood contaminants (Longnecker et al. 2003). Thus, the present study considered a substantial number of social and obstetric variables as cofactors, none of which affected our findings. Our results therefore suggest that recommendations on marine food during pregnancy should take into consideration the possible immunotoxic impact of the contaminants.

The observed associations with developmental exposures to suspected immunotoxicants must be evaluated in light of the increased vulnerability of the developing immune system (Dietert 2008). Because of the semiallogeneic pregnancy state, where graft rejection is suppressed, certain types of effects are more likely to be results of immunotoxicant exposures during the intrauterine developmental phase. During the early postnatal period, both immunosuppression and an increased risk of allergic disease can occur. The last-trimester fetus and the neonate usually exhibit comparatively depressed Th1-dependent functions, and current epidemiologic and experimental evidence on increased total IgE levels after exposure to various forms of stresses suggests that the postnatal acquisition of needed Th1 capacity could be a highly vulnerable target (Dietert 2008; Poulsen and Hummelshoj 2007). Accordingly, both dysfunction and misregulation are possible effects of developmental immunotoxicity (Dietert 2008).

Although breast-feeding appeared to be positively associated with the total IgE concentration, the adjustment for PCB exposure attenuated this association to a nonsignificant level. The possible impact of lactational immunotoxicant exposure, as reflected by the postnatal serum PCB concentrations in the present study, would suggest that associations between breast-feeding and serum IgE concentrations in children could, at least in part, be due to immunotoxic food contaminants transferred via human milk.

Current evidence is equivocal concerning the effect of breast-feeding on the child’s total serum IgE concentration. A prospective study in the United States found lower IgE concentrations at 8 years of age in breast-fed children, but only if the mother did not have an increased IgE level herself (Wright et al. 1999). Further, in 258 Pakistani children 6 months to 12 years of age, a total IgE concentration above a reference level occurred in about 80% of bottle-fed children and only in half as many of those that were breast-fed (Satwani et al. 2009). However, in 215 Polish children 8 months to 18 years of age, the duration of breast-feeding was not associated with the total IgE concentration (Daniluk et al. 2008).

Breast-feeding has often been considered a preventive factor in regard to allergy development, although some studies have suggested that breast-feeding may instead cause an increased risk (Bergmann et al. 2002; Kirsten 2009; van Odijk et al. 2003). Also, another Nordic study recently reported that longer breast-feeding was associated with a higher risk of atopic dermatitis but a lower risk of asthma (Giwercman et al. 2010). The conundrums of statistically significant associations in opposite directions in different populations may be attributable to the effects of one or more independent risk factors that differ between the populations studied. However, data on immunotoxicant exposures are not available from the studies on breast-feeding regarding allergy development or serum IgE concentrations. The present study indicates that an effect of breast-feeding per se is likely to be small and may be negligible. Adjustment for lactational exposures to the immunotoxicants would seem necessary to assess the true magnitude of an independent effect of breast-feeding on allergy risks.

The associations of PCB and methylmercury exposures with indicators of allergy and allergic disease may involve both stimulation and inhibition of immune system functions. Based on the exposure assessments at three or four occasions, prenatal and postnatal exposures seem to have different effects. For methylmercury, we observed exposure-associated effects only in relation to prenatal exposures, and the much lower postnatal exposures did not reveal any clear associations. However, PCB and methylmercury may well target different components of the immune system, and their effects would also depend on the stage of development. Ideally, immune system dysfunction should therefore not be assessed by means of a single or a few parameters nor at one stage of development only.

Our results may not necessarily be at odds with the “hygiene” hypothesis, which has been expressed in different terms regarding allergy and other diseases (Bach 2002). Rather, our data emphasize the need not to limit the focus only to gene–microbiome interactions but also to include environmental factors, such as immunotoxicants. Because our study provides evidence from a unique population with a well-characterized exposure to environmental chemicals from traditional food, the results provide insight into the potential effects of methylmercury and PCB exposures and their possible interaction with beneficial effects from breast-feeding. Even though the exposure in the Faroes may be less complex than elsewhere, the picture remains multifaceted.

Developmental immunotoxicity may predispose children to common diseases of increasing prevalence, such as childhood asthma and allergic diseases, and is therefore important from a public health perspective. Thus, our findings support the need for screening studies to identify immunotoxicants (Dietert 2008). In this regard, immunosuppression should not be considered as the only relevant outcome, and effects associated with developmental exposures need to be considered independently from effects in mature organisms. Because of uncertainty regarding interpretation of results from different animal models, human studies remain crucial, and prospective studies must incorporate delayed adverse outcomes of developmental exposures.

## Figures and Tables

**Table 1:** Characteristics of a Faroese birth cohort followed until 7 years of age (*n* = 464).

Variable	Result
Maternal age at parturition (years)	29.5 ± 6.3
Previous births (0/1/≥ 2) (%)	27.7/32.1/40.2
Smoking during pregnancy (no/yes) (%)	73.2/26.8
Alcohol consumption during pregnancy (never/ever) (%)	59.8/40.2
No. of fish dinners per week during pregnancy (≤ 1/2/3/≥ 4) (%)^a^	28.4/35.4/26.1/10.1
Gestational age (weeks)	39.8 ± 1.5
Birth weight (g)	3,722 ± 503
Sex (boys/girls) (%)	53.2/46.8
Maternal serum ∑PCB concentration (μg/g lipid)	1.25 (0.83–1.90)
Milk ∑PCB concentration (μg/g lipid)	1.34 (0.85–2.13)
Duration of exclusive breast-feeding (months)	4.6 ± 2.0
Total duration of breast-feeding (months)	9.8 ± 6.6
Child serum ∑PCB concentration (μg/g lipid),	
5 years of age	1.14 (0.70–1.93)
7 years of age	0.75 (0.43–1.38)
Mercury concentration	
Maternal hair (μg/g)	2.21 (1.3–4.1)
Cord blood (μg/L)	11.3 (7.4–21.0)
Child 5 years of age, blood (μg/L)	2.65 (1.35–5.4)
Child 7 years of age, blood (μg/L)	2.01 (1.01–4.3)
Age at 7-year examination (years)	7.53 ± 0.11

Values are mean ± SD, percent, or geometric mean (interquartile range).

a Data from 268 mothers only.

**Table 2:** Correlations between log-transformed exposure biomarkers for methylmercury and PCBs, and the duration of breast-feeding in a Faroese birth cohort followed prospectively until 7 years of age (*n* = 464).

	PCB		Mercury	
Exposure parameter	Prenatal	7 years	Prenatal	7 years
PCB				
Prenatal	1	0.52**	0.33**	0.22**
5 years	0.61**	0.80**	0.25**	0.23**
7 years	0.52**	1	0.27**	0.27**

Mercury				
Prenatal	0.33**	0.27**	1	0.28**
5 years	0.12*	0.24**	0.25**	0.52**
7 years	0.22**	0.27**	0.28**	1

Duration of breast-feeding				
Exclusive	0.02	0.41**	0.02	0.00
Total	−0.05	0.34**	−0.03	−0.01

* *p* < 0.01;

** *p* < 0.001.

**Table 3:** Exposure parameters [geometric mean (interquartile range)] for 464 birth cohort members examined at 7 years of age in tertile groups of serum total IgE concentrations.

	Serum total IgE concentration (kU/L)			
Exposure parameter	Low (< 13.6)	Medium (13.6–47.7)	High (> 47.7)	*p*-Value^a^
PCB (μg/g serum lipid)				
Prenatal	1.16 (0.73–1.79)	1.17 (0.76–1.97)	1.29 (0.83–2.3)	0.30
5 years	1.00 (0.54–1.85)	1.19 (0.76–1.91)	1.27 (0.81–2.2)	0.01
7 years	0.66 (0.35–1.30)	0.74 (0.46–1.26)	0.88 (0.56–1.47)	0.005

Mercury (μg/L blood)				
Prenatal	12.1 (6.6–19.4)	13.5 (7.8–21.7)	13.3 (7.6–24.7)	0.06
5 years	2.5 (1.26–4.9)	2.8 (1.41–6.1)	2.5 (1.41–4.4)	0.44
7 years	2.1 (0.95–4.5)	2.1 (1.07–4.2)	2.2 (1.06–4.9)	0.37

Duration of breast-feeding (months)				
Exclusive	4.2 (3–6)	4.8 (4–6)	5.0 (4–6)	0.003
Total	9.2 (5–12)	10.0 (7–12)	10.8 (7–13)	0.15

a For correlation of log-transformed variables (duration of breast-feeding not transformed).

**Table 4:** Serum grass-specific IgE concentrations in 464 birth cohort members at 7 years of age [geometric mean (interquartile range)].

	Serum grass-specific IgE concentration (kUA/L)		
Exposure parameter	Low (≤ 0.35; *n* = 425)	High (> 0.35; *n* = 39)	*p*-Value^a^
PCB (μg/g serum lipid)			
Prenatal	1.22 (0.79–2.0)	1.12 (0.54–2.3)	0.47
5 years	1.14 (0.69–1.94)	1.15 (0.81–2.3)	0.95
7 years	0.75 (0.43–1.39)	0.82 (0.61–1.43)	0.57

Methylmercury (μg/L)			
Prenatal	13.3 (7.6–22.5)	9.6 (6.2–12.2)	0.02
5 years	2.7 (1.35–5.2)	2.0 (1.29–3.0)	0.08
7 years	2.2 (1.05–4.5)	1.7 (0.76–3.0)	0.19

Duration of breast-feeding (months)			
Exclusive	4.6 (4–6)	5.0 (4–6)	0.23
Total	9.8 (6–12)	12.4 (8–14)	0.02

a For difference between groups (*t*-test).

**Table 5:** Exposure parameters and IgE results for 464 children with and without current or past history of asthma and/or atopic dermatitis by 5 or 7 years of age [average (interquartile range)].

	No allergy	Asthma		Atopic dermatitis	
Exposure parameter	(*n* = 378)	*n* = 35	*p*-Value^a^	*n* = 60	*p*-Value^a^
PCB (μg/g lipid)^b^					
Prenatal	1.24 (0.83–2.0)	1.46 (0.80–2.6)	0.17	0.70 (0.55–1.58)	0.01
5 years	1.17 (0.73–1.96)	1.40 (0.63–2.7)	0.20	0.56 (0.58–1.85)	0.07
7 years	0.77 (0.44–1.39)	0.89 (0.49–1.70)	0.33	0.60 (0.29–1.19)	0.04

Methylmercury (μg/L)^b^					
Prenatal	12.8 (7.2–21.1)	14.7 (7.6–29.2)	0.35	13.2 (7.4–24.2)	0.80
5 years	2.5 (1.34–4.7)	3.5 (1.69–10.2)	0.06	3.1 (1.41–6.7)	0.13
7 years	2.0 (1.00–4.6)	2.1 (1.06–3.9)	0.91	2.2 (1.07–4.6)	0.65

Duration of breast-feeding (months)					
Exclusive	4.5 (4–6)	5.0 (4–6)	0.14	4.5 (3–6)	0.92
Total	9.9 (6–12)	9.3 (7–12)	0.58	9.5 (6–12)	0.63

IgE at 7 years					
Total (kU/L)^b^	24.4 (9.3–63)	63.7 (24.3–243)	0.001	40.5 (15.3–136)	0.02
With grass-specific IgE > 0.35 kUA/L [*n* (%)]	19 (5)	8 (23)	0.14	15 (25)	< 0.001

a For comparison with subjects without allergy (*t*-test for continuous variables, chi-square test for categorical variables).

b Geometric mean.
